# SabaTracheid 1.0: A Novel Program for Quantitative Analysis of Conifer Wood Anatomy — A Demonstration on African Juniper From the Blue Nile Basin

**DOI:** 10.3389/fpls.2021.595258

**Published:** 2021-03-18

**Authors:** Eyob Gebrehiwot Gebregeorgis, Justyna Boniecka, Marcin Pia̧tkowski, Iain Robertson, Cyrille B. K. Rathgeber

**Affiliations:** ^1^Ethiopian Environment and Forest Research Institute, Addis Ababa, Ethiopia; ^2^Department of Plant Biology and Biodiversity Management, Addis Ababa University, Addis Ababa, Ethiopia; ^3^Department of Genetics, Faculty of Biological and Veterinary Sciences, Nicolaus Copernicus University, Toruń, Poland; ^4^Faculty of Mathematics and Computer Science, Nicolaus Copernicus University, Toruń, Poland; ^5^Department of Geography, Faculty of Science and Engineering, Swansea University, Swansea, United Kingdom; ^6^Université de Lorraine, AgroParisTech, INRAE, SILVA, Nancy, France

**Keywords:** quantitative wood anatomy (QWA), earlywood and latewood anatomy, dendroanatomy, tracheidogram methods, tropical conifers

## Abstract

Knowledge about past climates, especially at a seasonal time scale, is important as it allows informed decisions to be made to mitigate future climate change. However, globally, and especially in semi-arid Tropics, instrumental climatic data are scarce. A dendroclimatic approach may fill this gap, but tropical dendrochronological data are rare and do not yet provide fine resolution intra-annual information about past climates. Unlike in the Tropics, in the Mediterranean, temperate, alpine, and arctic regions, dendroanatomy and quantitative wood anatomy (QWA) are progressing fast attaining an intra-annual resolution, which allows a better understanding of seasonal climate dynamics and climate–growth relationships. The existing dendroanatomical and QWA methods aren’t suitable for tropical trees because they do not consider the high variation in tree ring width and the frequent occurrence of micro-rings containing only a few tracheids per radial file. The available tracheid analysis programs generally fail to provide multiple sectors for micro-rings and they are unable to compute most of the useful dendroanatomical parameters at fine temporal resolutions. Here, we present a program (SabaTracheid) that addresses the three main standard tasks that are necessary for QWA and dendroanatomy before running a climate analysis: (1) tracheidogram standardization, (2) sectoring, and (3) computing QWA and dendroanatomical variables. SabaTracheid is demonstrated on African Juniper (*Juniperus procera* Hochst. ex Endl), but it is potentially able to provide fine-resolution QWA and dendroanatomic data that could be used for dendroanatomical studies in all regions of the world. **SabaTracheid** is a freeware that quickly and accurately standardizes tracheidograms, divides tree rings into multiple regular sectors, computes useful dendroanatomic and QWA variables for the whole tree rings, early- and latewood portions, and each sector separately. This program is particularly adapted to deal with high inter-annual growth variations observed in tropical trees so that it assures the provision of complete sectoral QWA and dendroanatomical data for micro-rings as well. We demonstrate **SabaTracheid** using a dataset of 30 *Juniperus procera* tree rings from the Blue Nile basin, in Ethiopia. **SabaTracheid**’s ability to provide fine resolution QWA and dendroanatomic data will help the discipline develop in tropical as well as in the Mediterranean and temperate regions.

## Introduction

Understanding past-climate variability and its impact on vegetation is vital for assessing the influence of ongoing climatic change to adapt and mitigate its effects. In arid and semi-arid areas, climate change induced drought and seasonal shifts are affecting livelihoods. Thus, having a deep understanding of the past climate at a fine resolution of intra-annual pattern is crucial. Globally, instrumental climate records are characterized by low temporal and spatial resolution which calls for climate proxies from natural archives, preferably tree rings. Moreover, in the Tropics, where the instrumental climate record is inadequate, dendroclimatology is limited to annual or seasonal resolution (Worbes, 2002; Mokria et al., 2017; Gebregeorgis et al., 2020). However, finer temporal resolutions could be attained by extracting environmental information recorded in the dimensions of conifer wood cells, as it has been done for temperate and Mediterranean regions (Panyushkina et al., 2003; Liang et al., 2013; Hetzer et al., 2014; Castagneri et al., 2018; Piermattei et al., 2020). Dendroanatomy and quantitative wood anatomy (QWA) are fields of study aiming to support long-term intra-annual dendroclimatology (Panyushkina et al., 2003; Olano et al., 2012; Liang et al., 2013; Björklund et al., 2020; Lange et al., 2020). Hereafter, we define QWA as the measurement and analysis of tracheid anatomy and dendroanatomy as the analysis of the temporal relationship between wood anatomy and climate using QWA data. QWA and dendroanatomy compare anatomical features that are assumed to form in similar periods in different trees and years, in different tree rings of different sizes and numbers of cells, and between different tree rings (Vaganov, 1990; Olano et al., 2012). Thus, such studies require tracheidograms standardized into equal numbers of tracheids per radial file in a tree ring (Vaganov, 1990).

Few tracheid standardization techniques have been developed to analyze the different temporal resolution of tree rings. Existing methods are unable to consider the high intra-annual variation in tree ring width and frequent occurrence of micro rings. For instance, tree rings of *J. procera* are chracterised by 2–5 tracheids per radial row (Gebregeorgis et al., 2020). This variation is a characteristic feature of most tropical and some Mediterranean conifers suitable for dendrochronology (Gebregeorgis et al., 2020; Lange et al., 2020). Most dendroanatomical studies conducted in temperate or Mediterranean regions do sectoring prior to the measurement and standardization of tracheids (Castagneri et al., 2017a; Lange et al., 2020). However, in the case of micro-rings, this may eventually result in missing dendroanatomical values for some sectors (Lange et al., 2020). Sectoring after standardization could increase the probability that tracheids formed at similar time in different years will be grouped in the same percentile position or sectors.

Although accurate demarcation of earlywood (EW) to latewood (LW) transition has vital assistance toward obtaining a fine temporal resolution of dendroanatomical and QWA information (Campelo et al., 2016), there has only been limited success in developing an accurate species-specific earlywood to latewood transition threshold. Mork’s index (Denne, 1988) is one of the most widely used techniques in the case of earlywood to latewood transition boundary demarcation (Park et al., 2006). However, Mork’s index shows very few or no latewood cells in many conifers, especially the tropical ones, though the borders could visually be identified through finding the points where observed gradual decline in lumen diameter (LD) and increase in double wall thickness (WT) in the latewood of tree rings occurred.

Following the advancement in microscopic slide preparation (Gärtner and Schweingruber, 2014; von Arx et al., 2016), accompanied with high resolution imaging and measurement techniques, and softwares [e.g., WinCELL (Larocque and Smith, 2005), ImageJ (Schuldt et al., 2013), and ROXAS (von Arx and Carrer, 2014)], dendroanatomy and QWA has improved remarkably. Recent dendroanatomy and QWA programs (Campelo et al., 2016; von Arx et al., 2016; Ziaco, 2020) have contributed to the realization of successful fine-resolution tracheid chronologies and dendroclimatic studies (Carrer et al., 2016, 2018; Castagneri et al., 2018; Belokopytova et al., 2019; Lange et al., 2020). However, most available programs are limited to tracheid measurement (Dyachuk et al., 2020) and standardization (Campelo et al., 2016; Ziaco, 2020) only. This solely is not enough towards developing QWA and dendroanatomy since programs that further analyze the measured and standardized series and generate sectoral values are needed.

The program presented here, **SabaTracheid**, has a set of functions for fast, simple, and accurate computation of dendroanatomical and QWA parameters throughout whole tree rings, and over different portions of tree rings (Figure 3). Once the transition from earlywood to latewood is visually identified and marked, data imported into **SabaTracheid** and processed can serve to generate an observation based species-specific lumen diameter to double wall thickness ratio (LD/WT) threshold. Threshold values calculated in this way could be introduced into automated tracheid measurement programs [e.g., WinCELL (Larocque and Smith, 2005) and ROXAS (von Arx and Carrer, 2014)], to improve the future detection of the transition boundary. This novel calculation of the transition boundary may be applicable to conifers that have insufficient latewood cells according to Mork’s index (Park et al., 2006). However, in the cases where manual demarcation of the earlywood to latewood boundary is impossible, SabaTracheid has an option of demarcating the boundary using Mork’s index.

The core novel approach of **SabaTracheid** is that it applies a standardization technique which accommodates the characteristics of trees from arid and semi-arid regions. This is achieved by replicating tracheids of narrow tree rings (Vaganov, 1990), and then a relative positioning method (Campelo et al., 2016), prior to sectoring tree rings. Moreover, the program provides fine temporal resolution of anatomical series by sectoring tracheidograms into multiple sectors per radial file. In this article, the program is demonstrated using tracheid measurement data from African Juniper tree rings from the Upper Blue Nile basin, Ethiopia (Gebregeorgis et al., 2020).

## Materials and Methods

### Presentation of the Training Dataset

**FIGURE 1:**
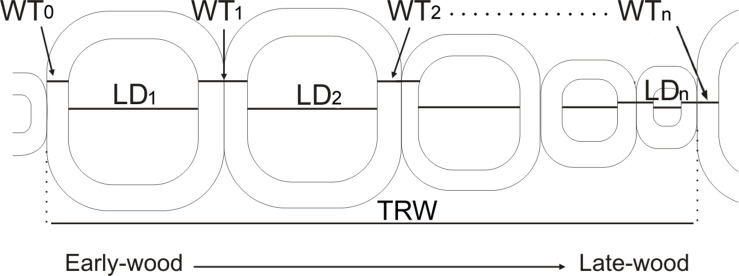
A demonstration of tracheid parameters measured and imported into SabaTracheid. WT_0_, single wall thickness of the cell at the start of a tree ring; LD_1_, lumen diameter of the first cell in a tree ring; WT_1_, double wall thickness between first and second cells in a tree ring; LD_2_, lumen diameter of the second cell in a tree ring; WT_2_, double wall thickness between second and third cells; LD_n_, lumen diameter of the last cell in a tree ring; WT_n_, double wall thickness between the last cell in the analyzed tree ring and the first cell in the next tree ring; TRW, tree ring width.

**FIGURE 2:**
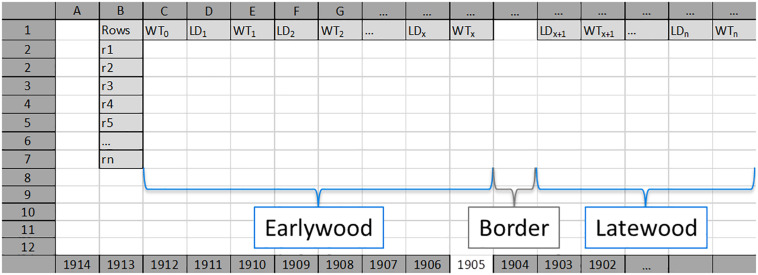
An example of an Excel input worksheet for each tree ring (here 1905). Earlywood values for each radial row (Rows; from r1 to rn) start with single wall thickness values of first tracheids (WT_0_) per row, and are followed by alternating lumen diameter (LD, LD_1_–LD_x_) and double wall thickness (WT, WT_1_–WT_x_) values. Earlywood and latewood are separated by an empty column (named as “Border”). Latewood values for each radial row start with lumen diameter value of the first tracheid (LD_x+__1_) after the border, and are followed by alternating double wall thickness and lumen diameter values. LD_x_, last LD value of earlywood; WT_x_, last WT value of earlywood; LD_x+__1_, first LD value of latewood; WT_x+__1_, first WT value of latewood. LD_n_, lumen diameter of the last cell in the tree ring; WT_n_, double wall thickness between the last cell in the analyzed tree ring and the first cell in the next tree ring.

**SabaTracheid**’s functions were demonstrated using tracheid LD and WT measurement data from 30 tree rings (AD 1901–1930) of an absolutely dated core sample of *Juniperus procera* from Gonder Qusquam church, Ethiopia, that was confirmed by radiocarbon dating (Gebregeorgis et al., 2020). Firstly, measured values of tracheid LD and WT (Figure 1) for five to eight radial rows per tree ring were entered into Excel spreadsheets named after each tree ring (e.g., 1905; Figure 2).

### Input File Preparation

**SabaTracheid** can import and process tracheid measurement data generated by any means of measurement as long as it is prepared in either of the two specific Excel sheet forms as is demonstrated (Figure 2 and Supplementary Figure 1). For more detailed clarification, in addition to Figure 2 and Supplementary Figure 1, the two actual input files are presented as Supplementary Files 1 and 3. Regarding the earlywood to latewood transition boundary, SabaTracheid has two options between which one can choose by tick marking in the check-box depending on the input file structure. The first option is that the border, which was visually identified and manually marked during tracheid measurement, was demarcated by inserting an empty column regardless of variation in number of cells between rows of a tree ring (Figure 2 and Supplementary File 1). Here, we should note that the program will only identify one such straight column per sheet to be the border between the EW and LW of each radial file. The second option is that the input file (Supplementary Figure 1 and Supplementary File 3) is prepared without manual marking as the program has a check box option “Mork’s latewood” which enables us to identify the boundary using Mork’s index (Denne, 1988). According to Mork’s definition, a latewood cell is one that the value of twice the tracheid’s double cell wall thickness divided by cell lumen exceeds 1 (Denne, 1988). Then, the rest of the computation will remain the same as that for the first input type. It should be noted that preparation of the input files could be time-consuming unless done with R codes.

The Excel workbooks (files), comprising sheets containing each tree ring’s tracheid values of five to eight rows (Figure 2 and Supplementary File 1), were then imported into **SabaTracheid**. To ease extraction of results and to avoid confusion, we suggest preparing separate Excel files for individual core samples. Importantly, **SabaTracheid** is designed to compute data from unlimited radial files of tracheids per tree ring [Rows; from r1 to rn (Figure 2 and Supplementary Figure 1)].

### Dendroanatomical and QWA Variable Computation

**FIGURE 3:**
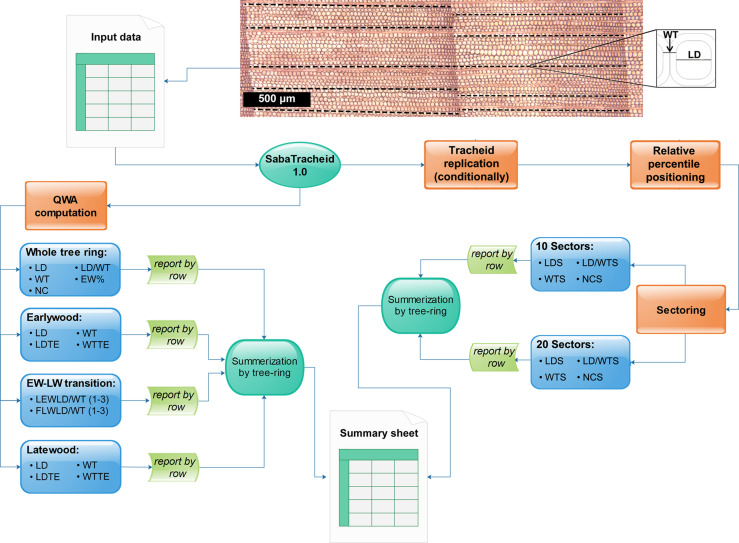
Schematic summary of all stepwise computations of tracheid variables in **SabaTracheid 1.0**. The image and Excel file “Input data” are the tracheid image and the input file, followed by computation of quantitative wood anatomical (QWA) parameters prior standardization, i.e. mean values of lumen diameter (LD), double wall thickness (WT), number of cells (NC), ratio of LD to WT (LD/WT), earlywood percentage (EW%), lumen diameter excluding transition cells (LDTE), double wall thickness excluding transition cells (WTTE), the last three earlywood cells’ LD/WT [LEWLD/WT (1–3)], and the first three latewood cells’ LD/WT [FLWLD/WT (1–3)], then summarized per tree ring at different sections of the ring. The second step begins with standardization by cell values multiplication (if needed) and computation of the relative position of tracheid’s centers over their respective tree ring widths. The standardized tracheidogram is sectored into 10 and (optionally) 20 equal parts, and then lumen diameter (LDS), double wall thickness (WTS) and ratio of lumen diameter to double wall thickness (LD/WTS), and number of tracheids (NCS) per each sector are computed.

**SabaTracheid** begins analysis by computing various dendroanatomical and QWA parameters (Figure 3). Calculations are performed for:

• The entire tree ring [computes mean LD and WT values, counts number of cells (NC), computes LD/WT ratio, estimates earlywood proportion (EW%) on non-standardized row data].
• Earlywood [computes means of earlywood LD, LD excluding transitional cells (LDTE), WT, and double wall thickness excluding transitional cells (WTTE) on non-standardized row data].
• At the transition between earlywood and latewood, SabaTracheid estimates the last three earlywood tracheid’s ratio of lumen diameter to double wall thickness [LEWLD/WT (1–3)] and the first three latewood tracheid’s ratio of lumen diameter to double wall thickness [FLWLD/WT (1–3)] on non-standardized tracheidograms.
• Latewood [computes means of latewood LD, LD excluding transitional cells (LDTE), WT, and double wall thickness excluding transitional cells (WTTE) on non-standardized row data].

The LEWLD/WT (1–3) and FLWLD/WT (1–3) outputs can be used in further analysis to test the statistical significance of the manually demarcated EW-LW transition, based on e.g., ANOVA and Tukey’s statistical tests. The number of tree rings that showed latewood cells according to Mork’s index and the manual demarcation was compared using the two types of the input files (Supplementary Files 1 and 2).

### Tracheidogram Standardization

Tracheids of different tree rings need to be standardized in order to be able to compare tree rings of varying width. Three different standardization techniques were to some extent utilized in SabaTracheid. The first technique stretches narrow rings and shrinks wide rings in order to overlay and compare tree rings with a varying number of cells by limiting their cells to certain number per radial file (Vaganov, 1990). The second technique standardizes tree ring cells using the relative positioning of each tracheid’s center which ensures better overlaying and thereby comparison of different tree rings of differing size as well as cell numbers formed in similar times of different years (Campelo et al., 2016). The third technique enables grouping/sectoring of cells according to their relative percentile position in their radial rows (Castagneri et al., 2017a). The standardization and sectoring of the *Juniperus procera* tracheids was performed by adopting the following three steps. Firstly, as *Juniperus procera* has small tracheids, the chances of having missing values in any sector were minimized by replicating tracheids, if there were less than 40 tracheids per row, until they attained a minimum of 40 tracheids per row (Vaganov, 1990). Any changes are recorded in the Summary Sheet output of the software (Supplementary Files 2, 4). For example, if a row has only 5 tracheids, values of each variable (wall thickness on the left side of a cell, lumen diameter and wall thickness on right side of a cell) are replicated at least eight times to eventually obtain values for at least 40 cells. Secondly, the relative position of each tracheid’s center over the widths of their respective tree rings was computed (Campelo et al., 2016). For instance, the relative position of a second tracheid in a radial row is calculated as follows: the single wall thickness value of the first tracheid (WT_0_) in the row plus value of the lumen diameter of the first cell in the row (LD_1_) plus double wall thickness between lumens of the first and second cells in the row (WT_1_) plus half of the second tracheid’s lumen diameter (LD_2_), all divided by the tree ring width, and the result multiplied by 100. Thirdly, in order to get fine intra-annual tracheidal chronologies, grouping of tracheids into equidistant sections, i.e. sectors, was important (Castagneri et al., 2017b).

### Tracheidogram Sectoring

Radial values of lumen diameter sector (LDS), double wall thickness sector (WTS), and ratio of lumen diameter to double wall thickness sector (LD/WTS) were averaged at every 5% and optionally at every 10% of relative position, providing 20 and 10 sectors of tracheid variables, respectively (Castagneri et al., 2017b). The novelty of our method, making it unique in the literature, is that, unlike other methods that sector tree rings before any tracheid measurement (Hetzer et al., 2014; Castagneri et al., 2017b, 2018), **sectoring is done after standardizing tracheidograms.** This is because variation in the sizes of different tree rings, and that between different rows of cells within a tree ring, require standardization to increase the chance that tracheids that must have formed at a similar time of different years will be grouped into the same percentile position and or sectors. **Thus, it has the potential of being a global tracheid analysis tool.**

For the demonstration of SabaTracheid, the computation of all the variables in the 30 Excel spreadsheets in the input file took approximately eight seconds using a standard computer equipped with a 2.2 GHz dual core processor. Importantly, if the data in the input files were incorrectly formatted, **SabaTracheid** displays an error messages that specifies the problematic cell in the input Excel spreadsheet and the reason for the error.

### Software Availability

**SabaTracheid** is a Java based program which is freely downloadable for use from GitHub repository (10.6084/m9.figshare.12612128).

## Results

**SabaTracheid**’s output file, i.e. an Excel workbook, contains an individual worksheet for each tree ring file. Each worksheet displays radial LD, NC, WT, and multiple dendroanatomical and QWA parameter values for each radial row file in each tree ring, relative positions of each tracheid’s center (1–100%), and sectoral values (every 5% or 10%) (Supplementary Files 2 and 4). The means of all listed tracheid parameters averaged per tree ring are included in the summary sheet of the output workbook (Figure 3 and Supplementary Files 2 and 4).

**FIGURE 4:**
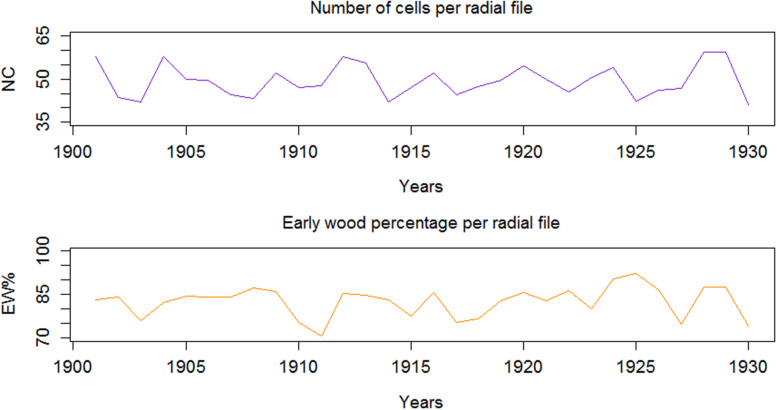
Inter-annual variability of number of cells (NC), and earlywood proportion (EW%) per radial file of tree rings.

Considering examples from QWA parameters, i.e. EW% and NC, computed for the 30 tree rings (AD 1901-1930) of *Juniperus procera* using **SabaTracheid**, the NC varied from 5 to 76 and averaged 53, while EW% averaged 91.7% (Figure 4). These results may be used to calculate tree hydraulic conductivity, to study tree physiology, drought adaptation strategies of trees, and to obtain many dendroclimatological parameters.

**FIGURE 5:**
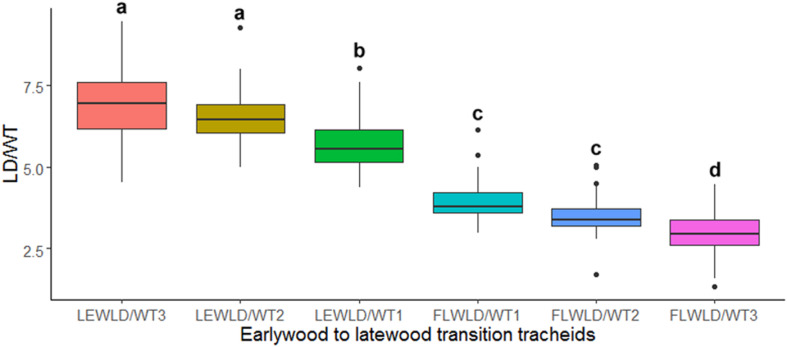
Box plot of mean tracheid lumen diameter to double wall thickness ratio (LD/WT) of the last three earlywood [LEWLD/WT (1–3)] and first three latewood [FLWLD/WT (1–3)] tracheids of 30 *Juniperus procera* tree rings dated 1901–1930 AD. Different letters indicate statistically significant differences in the measured parameter between the tested tracheids (*p* < 0.01).

The ANOVA and Tukey’s test on LD/WT ratio values of the manually demarked earlywood to latewood transition cells, obtained from SabaTracheid’s outputs, showed a statistically significant decline in LD/WT ratio of the last earlywood [LEWLD/WT1] and the first latewood [FLWLD/WT1] tracheids of the 30 *Juniperus procera* tree rings (Figure 5). These variables can give us an insight of how abrupt EW-LW transition could be for each species. Once computed for a species, an EW-LW transition factor could be developed and implemented in automatic border identification programs.

Amongst the 30 *Juniperus procera* tree rings considered in this study, only 11 of them showed to have latewood cells that qualified for Mork’s definition (Supplementary File 4).

**FIGURE 6:**
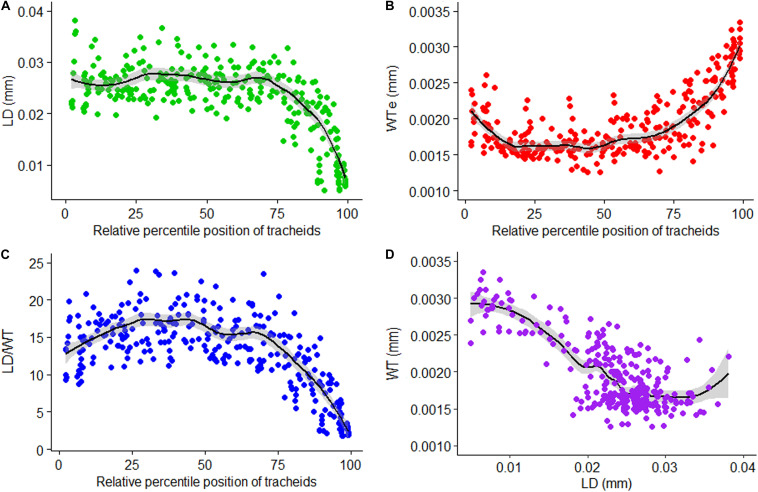
Relative position standardized tracheidogram of lumen diameter (LD) **(A)**, single wall thickness of each cell (WTe) **(B)**, lumen diameter to double wall thickness ratio (LD/WT) **(C)**, and a scatterplot of wall thickness versus lumen diameter **(D)**.

LOESS regression curves fitted on graphical presentations of standardized values of LD (Figure 6A), single wall thickness of each cell (WTe; Figure 6B), and LD/WT (Figure 6C) before sectoring, and non-standardized values of radial LD and WT (Figure 6D), are plotted (Figure 6). The standardized tracheidal parameters showed intra-annual pattern which calls for sectoring them along their relative percentile positions. This may eventually give us an intra-annual climate record and other environmental signals preserved in them.

**FIGURE 7:**
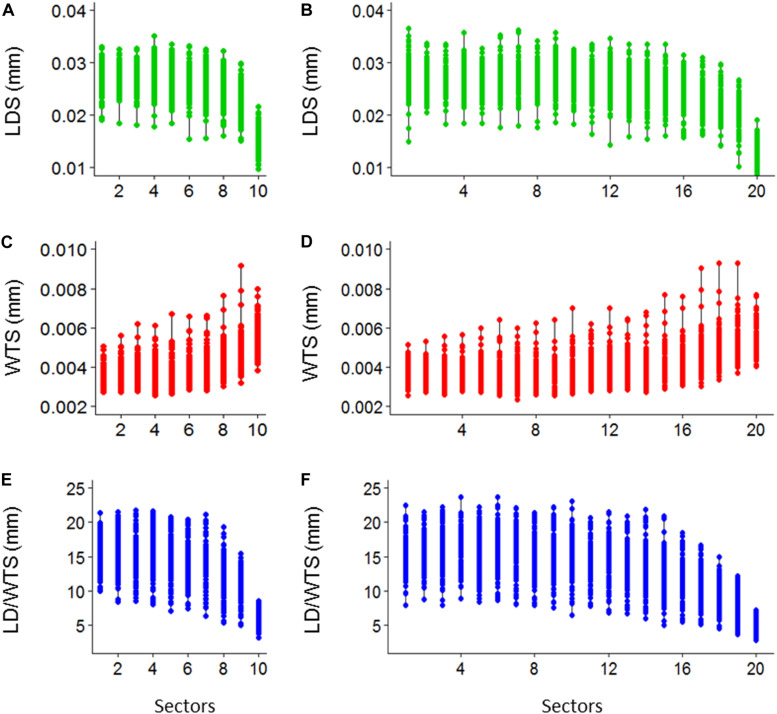
Demonstrative plot of 10 sectoral mean values of lumen diameter (LDS), double wall thickness (WTS) and lumen diameter to double wall thickness ratio (LD/WTS) (**A–C**, respectively), and 20 sectoral mean values of LDS, WTS, and LD/WTS (**D–F**, respectively), of SabaTracheid’s output for *Juniperus procera* trees.

Ten sectoral values per tree ring of standardized LDS, WTS, and LD/WTS are obtained (Figures 7A–C) and, similarly, the twenty sectoral values are also presented (Figures 7D–F).

## Discussion

**SabaTracheid** facilitates rapid and accurate standardization of tracheid measurements through a combination of a few standardization techniques, and computes multiple dendroanatomical and QWA parameters for multiple tree rings (Figure 3). Dendroanatomical variables are the tracheid variables computed from standardized and sectored tracheids of dated tree rings (Figure 3). QWA parameters are those mostly computed from the non-standardized tracheids (Figure 3). The output file contains detailed computation of each parameter for each set of radial rows within a tree ring and data for each ring is outputted to a separate worksheet (Supplementary Files 2 and 4). It also provides a summary sheet per workbook to ease extraction of outputs (Supplementary Files 2 and 4). Developing such programs may strengthen and diversify proxies by considering NC, LD, and other dendroanatomical and QWA parameters that could be integrated well with dendrochronology. It contributes toward more fully understanding the sensitivity of trees to climate (Olano et al., 2012; Piermattei et al., 2020).

**SabaTracheid** enabled the computation of multiple QWA and dendroanatomical parameters for hundreds of tree rings in a few seconds (Supplementary Files 2, 4 and Figure 3). The analysis of proportions and relationships between QWA parameters, such as EW%, NC, and their relation to tree ring width gives insight into the drought adaptation mechanism of a tree, and the structural function and properties of its wood (Olano et al., 2012). This ultimately facilitates understanding of the environmental conditions responsible for these parameters’ values (Domec and Gartner, 2002; Oribe et al., 2003; Kennedy, 2010; Martin-Benito et al., 2013).

**SabaTracheid** may help to develop an observation-based species-specific earlywood to latewood boundary threshold of LD to WT ratio (Figure 5). This is crucial in intra-annual climate-growth relationship research since the two sections of wood form at different seasons of the year (Camarero et al., 2010). As demonstrated by Campelo et al. (2016), relative positioning using grayscales showed 10% higher earlywood proportion than two other methods employed on the same sample tree rings. Thus, attention to choice of standardization method might determine the resolution and quality of seasonal environmental signals obtained in the field of dendroanatomy. This is one of the rationales behind combining several existing tracheid standardization techniques into **SabaTracheid**. The manual demarcation used in this study demonstrated that the entire 30 tree rings possess latewood cells while when using Mork’s index only 11 of them fulfilled this requirement (Supplementary Files 2 and 4). Consequently, the manual demarcation of the earlywood to latewood boundary, based upon the measurement of *Juniperus procera*’s tracheids, is a better measure of the transition than Mork’s index. Thus, we recommend the computation of earlywood to latewood transition threshold for each species to enable the application of automated programs for QWA and dendroanatomical studies (Peters et al., 2018; Ziaco, 2020).

**SabaTracheid** has combined different tracheidogram standardization techniques, i.e. the idea of stretching narrow tree rings (micro-rings) through replication of their tracheids’ values until they attain the minimum number of tracheids per tree ring (Vaganov, 1990), relative positioning to compare anatomical features of different rings that have most likely formed in comparable seasons of different years (Campelo et al., 2016) and tree ring sectoring which groups tracheids according to their relative percentile positions (Castagneri et al., 2017a), but, importantly, it standardizes tracheidograms prior to sectoring. Sectoring *Juniperus procera* tracheidograms after tracheid measurement and standardization may increase the efficiency of distributing tracheid variables of a tree ring into equivalent parts despite their differing cell counts and ring widths (Figures 6, 7). Moreover, it may help to group tracheids of different years that have most likely formed at similar times of different years (Ziaco, 2020). This may be highly important, mainly for tropical conifers, which vary greatly in inter-annual tree ring width and cell number. On the other hand, previous sectoral chronological studies have sectored tree rings before measuring tracheids and proceeded to standardization (Castagneri et al., 2018; Lange et al., 2020). Failing to stretch narrow micro-rings through standardization may eventually result in missing values of tracheids in certain sectors. However, studies that sectored before standardization eventually provided anatomical series that were later compared against finer-temporal-resolution climate studies (Hetzer et al., 2014; Castagneri et al., 2017a, 2018; Lange et al., 2020).

Standardizing tracheidograms of *Juniperus procera* from tropical east Africa using relative positioning in a tree ring (Figures 6 and 7) seems to help efficiently prepare data for further analysis to extract climatic and tree-growth signals from tracheids. Similar results were obtained while comparing different tracheid standardization methods on Mediterranean conifers (Campelo et al., 2016). However, no similar study exists for tropical conifers.

The fact that **SabaTracheid** is a Java program makes it compatible with most computer operating systems and easy to use. It is freely downloadable at 10.6084/m9.figshare.12612128 and requires no specialized installation procedure. Moreover, it provides update notifications. The input file from direct or automated measurement should be carefully prepared, as it determines the next steps in analysis of tracheid data. Importantly, even with incorrect input data file preparation, the error notification in **SabaTracheid** guides the user to find possible errors in particular worksheet cells. The fact that the input and output files are Excel files eases the task of exporting and further analysis of results.

We believe that **SabaTracheid** may be useful for intra-annual dendroclimatology and dendroanatomical studies of any region as it uses a combination of standardization techniques proven (to give reliable results) on samples characterized by frequent occurrence of micro-rings, wedging rings and missing rings. Although other similar function packages in R have recently been reported (Peters et al., 2018; Ziaco, 2020), they mostly focus upon standardizing tracheidograms, so do not compute other dendroanatomical and QWA parameters. Our ambition is that this freeware will help expand the temporal and spatial resolution of dendroanatomy and be a global tracheid analysis tool, because it expands intra-annual dendroclimatology to tropical and other regions. It is user friendly freeware which doesn’t require programming skill, it could be used by researchers that are at different levels of expertise. This way, we may increase the global coverage and reproducibility of intra-annual dendroclimatic studies.

## Data Availability Statement

The raw data supporting the conclusions of this article will be made available by the authors, without undue reservation.

## Author Contributions

EG has participated in the conception of the original idea, designing of the methods and the program, manuscript write-up, editing, and correspondence. JB has participated in designing the method and the program, participated in editing the manuscript. MP took part in the implementation of the designed SabaTracheid 1.0 program in Java and in editing the manuscript. IR coordinated obtaining the samples as well as editing and improving the manuscript. CR has evaluated, improved the contents, validated, and has significantly improved the manuscript. All authors contributed to the article and approved the submitted version.

## Conflict of Interest

The authors declare that the research was conducted in the absence of any commercial or financial relationships that could be construed as a potential conflict of interest. The handling editor declared a past co-authorship with one of the authors CR.

## Supplementary Material

Supplementary Figure 1 An example of SabaTracheid’s second option Excel input worksheet for each tree ring (here 1905). Radial rows values (Rows; from r1 to rn) start with single wall thickness values of first tracheids (WT_0_) per row, and are followed by alternating lumen diameter (LD; LD_1_-LD_n_) and double wall thickness (WT; WT_1_-WT_n_) values. LD_n_, lumen diameter of the last cell in the tree ring; WT_n_, double wall thickness between the last cell in the analyzed tree ring and the first cell in the next tree ring. TRW, tree ring width.

## References

[B1] Belokopytova L. V. Babushkina E. A. Zhirnova D. F. Panyushkina I. P. Vaganov E. A. (2019). Pine and larch tracheids capture seasonal variations of climatic signal at moisture-limited sites. *Trees Struct. Funct.* 33 227–242. 10.1007/s00468-018-1772-2

[B2] Björklund J. Seftigen K. Fonti P. Nievergelt D. von Arx G. (2020). Dendroclimatic potential of dendroanatomy in temperature-sensitive *Pinus sylvestris*. *Dendrochronologia* 60:125673. 10.1016/j.dendro.2020.125673

[B3] Camarero J. J. Olano J. M. Parras A. (2010). Plastic bimodal xylogenesis in conifers from continental mediterranean climates. *New Phytol.* 185 471–480. 10.1111/j.1469-8137.2009.03073.x 19895415

[B4] Campelo F. Nabais C. Carvalho A. Vieira J. (2016). tracheideR-an R package to standardize tracheidograms. *Dendrochronologia* 37 64–68. 10.1016/j.dendro.2015.12.006

[B5] Carrer M. Brunetti M. Castagneri D. (2016). The imprint of extreme climate events in century-long time series of wood anatomical traits in high-elevation conifers. *Front. Plant Sci.* 7:683. 10.3389/fpls.2016.00683 27242880 PMC4870858

[B6] Carrer M. Unterholzner L. Castagneri D. (2018). Wood anatomical traits highlight complex temperature influence on *Pinus cembra* at high elevation in the Eastern Alps. *Int. J. Biometeorol.* 62 1745–1753. 10.1007/s00484-018-1577-4 29961923

[B7] Castagneri D. Battipaglia G. von Arx G. Pacheco A. Carrer M. (2018). Tree-ring anatomy and carbon isotope ratio show both direct and legacy effects of climate on bimodal xylem formation in *Pinus pinea*. *Tree Physiol.* 38 1098–1109. 10.1093/treephys/tpy036 29688500

[B8] Castagneri D. Fonti P. Von Arx G. Carrer M. (2017a). How does climate influence xylem morphogenesis over the growing season? Insights from long-term intra-ring anatomy in *Picea abies*. *Ann. Bot.* 119 1011–1020. 10.1093/aob/mcw274 28130220 PMC5604563

[B9] Castagneri D. Regev L. Boaretto E. Carrer M. (2017b). Xylem anatomical traits reveal different strategies of two mediterranean oaks to cope with drought and warming. *Environ. Exp. Bot.* 133 128–138. 10.1016/j.envexpbot.2016.10.009

[B10] Denne M. P. (1988). Definition of latewood according to Mork (1928). *IAWA Bull.* 10 59–62. 10.1163/22941932-90001112

[B11] Domec J.-C. Gartner B. L. (2002). How do water transport and water storage differ in coniferous earlywood and latewood? *J. Exp. Bot.* 53 2369–2379. 10.1093/jxb/erf100 12432029

[B12] Dyachuk P. Arzac A. Peresunko P. Videnin S. Ilyin V. Assaulianov R. (2020). AutoCellRow (ACR) – a new tool for the automatic quantification of cell radial files in conifer images. *Dendrochronologia* 60:125687. 10.1016/j.dendro.2020.125687

[B13] Gärtner H. Schweingruber F. (2014). Microscopic preparation techniques for plant stem analysis. *Tree-ring Res.* 70, 55–56.

[B14] Gebregeorgis E. G. Robertson I. Koprowski M. Zhou L. P. Gao P. Williams A. P. (2020). Historical droughts recorded in extended *Juniperus procera* ring-width chronologies from the Ethiopian Highlands. *Int. J. Biometeorol.* 64 739–753. 10.1007/s00484-020-01863-7 32008098 PMC7220890

[B15] Hetzer T. Bräuning A. Leuschner H. H. (2014). High-resolution climatic analysis of wood anatomical features in Corsican pine from Corsica (France) using latewood tracheid profiles. *Trees Struct. Funct.* 28 1279–1288. 10.1007/s00468-014-1045-7

[B16] Kennedy C. (2010). *Dendroclimatology of Picea glauca at Tree Line in Northern Labrador, Canada.* Masters thesis, St. John’s, NL: Memorial University of Newfoundland.

[B17] Lange J. Carrer M. Pisaric M. F. J. Porter T. J. Seo J. Trouillier M. (2020). Moisture-driven shift in the climate sensitivity of white spruce xylem anatomical traits is coupled to large-scale oscillation patterns across northern treeline in northwest North America. *Glob. Chang. Biol.* 26 1842–1856. 10.1111/gcb.14947 31799729

[B18] Larocque S. J. Smith D. J. (2005). A dendroclimatological reconstruction of climate since AD 1700 in the Mt. Waddington area, British Columbia Coast Mountains, Canada. *Dendrochronologia* 22 93–106. 10.1016/j.dendro.2005.02.003

[B19] Liang W. Heinrich I. Simard S. Helle G. Liñán I. D. Heinken T. (2013). Climate signals derived from cell anatomy of Scots pine in NE Germany. *Tree Physiol.* 33 833–844. 10.1093/treephys/tpt059 23999138

[B20] Martin-Benito D. Beeckman H. Cañellas I. (2013). Influence of drought on tree rings and tracheid features of *Pinus nigra* and *Pinus sylvestris* in a mesic mediterranean forest. *Eur. J. For. Res.* 132 33–45. 10.1007/s10342-012-0652-3

[B21] Mokria M. Gebrekirstos A. Abiyu A. Van Noordwijk M. Bräuning A. (2017). Multi-century tree-ring precipitation record reveals increasing frequency of extreme dry events in the upper Blue Nile River catchment. *Glob. Chang. Biol.* 23 5436–5454. 10.1111/gcb.13809 28712116

[B22] Olano J. M. Eugenio M. García-Cervigón A. I. Folch M. Rozas V. (2012). Quantitative tracheid anatomy reveals a complex environmental control of wood structure in continental mediterranean climate. *Int. J. Plant Sci.* 173 137–149. 10.1086/663165

[B23] Oribe Y. Funada R. Kubo T. (2003). Relationships between cambial activity, cell differentiation and the localization of starch in storage tissues around the cambium in locally heated stems of *Abies sachalinensis* (Schmidt) Masters. *Trees* 17 185–192. 10.1007/s00468-002-0231-1

[B24] Panyushkina I. P. Hughes M. K. Vaganov E. A. Munro M. A. (2003). Summer temperature in northeastern Siberia since 1642 reconstructed from tracheid dimensions and cell numbers of *Larix cajanderi*. *Can. J. For. Res.* 33 1905–1914. 10.1139/x03-109

[B25] Park Y.-I. (David) Dallaire G. Morin H. (2006). A method for multiple intra-ring demarcation of coniferous trees. *Ann. For. Sci.* 63 9–14. 10.1051/forest:2005093

[B26] Peters R. L. Balanzategui D. Hurley A. G. von Arx G. Prendin A. L. Cuny H. E. (2018). RAPTOR: row and position tracheid organizer in R. *Dendrochronologia* 47 10–16. 10.1016/j.dendro.2017.10.003

[B27] Piermattei A. von Arx G. Avanzi C. Fonti P. Gärtner H. Piotti A. (2020). Functional relationships of wood anatomical traits in Norway Spruce. *Front. Plant Sci.* 11:683. 10.3389/fpls.2020.00683 32528514 PMC7266088

[B28] Schuldt B. Leuschner C. Brock N. Horna V. (2013). Changes in wood density, wood anatomy and hydraulic properties of the xylem along the root-to-shoot flow path in tropical rainforest trees. *Tree Physiol.* 33 161–174. 10.1093/treephys/tps122 23292668

[B29] Vaganov E. A. (1990). “Basic chronology statistics and assesment,” in *Methods of Dendrochronology: Applications in the Environmental Sciences*, eds Cook E. R. Kairiukstis L. A. (Dordrecht: Springer), 137–152. 10.1007/978-94-015-7879-0

[B30] von Arx G. Carrer M. (2014). ROXAS-a new tool to build centuries-long tracheid-lumen chronologies in conifers. *Dendrochronologia* 32 290–293. 10.1016/j.dendro.2013.12.001

[B31] von Arx G. Crivellaro A. Prendin A. L. Èufar K. Carrer M. (2016). Quantitative wood anatomy—practical guidelines. *Front. Plant Sci.* 7:781. 10.3389/fpls.2016.00781 27375641 PMC4891576

[B32] Worbes M. (2002). One hundred years of tree-ring research in the tropics – a brief history and an outlook to future challenges. *Dendrochronologia* 20 217–231. 10.1078/1125-7865-00018

[B33] Ziaco E. (2020). A phenology-based approach to the analysis of conifers intra-annual xylem anatomy in water-limited environments. *Dendrochronologia* 59:125662. 10.1016/j.dendro.2019.125662

