# Supplementary figures and images for: SabaTracheid 1.0: A Novel Program for Quantitative Analysis of Conifer Wood Anatomy — A Demonstration on African Juniper From the Blue Nile Basin
Source: Front Plant Sci. 2021 Mar 18;12:595258. doi: 10.3389/fpls.2021.595258 (PMC8013728; doi:10.3389/fpls.2021.595258)

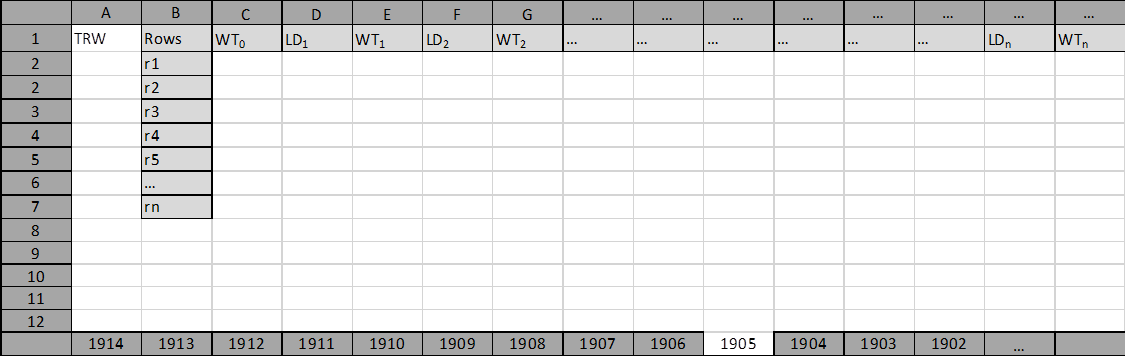

Supplement: Supplementary Figure 1 — An example of SabaTracheid’s second option Excel input worksheet for each tree ring (here 1905). Radial rows values (Rows; from r1 to rn) start with single wall thickness values of first tracheids (WT0) per row, and are followed by alternating lumen diameter (LD; LD1-LDn) and double wall thickness (WT; WT1-WTn) values. LDn, lumen diameter of the last cell in the tree ring; WTn, double wall thickness between the last cell in the analyzed tree ring and the first cell in the next tree ring. TRW, tree ring width. [file Image_1.tif]
